# Fat Mass Is Positively Associated with Estimated Hip Bone Strength among Chinese Men Aged 50 Years and above with Low Levels of Lean Mass

**DOI:** 10.3390/ijerph14040453

**Published:** 2017-04-24

**Authors:** Guiyuan Han, Yu-Ming Chen, Hua Huang, Zhanyong Chen, Lipeng Jing, Su-Mei Xiao

**Affiliations:** Department of Medical Statistics and Epidemiology, School of Public Health, Sun Yat-Sen University, Guangzhou 510080, China; hangy3@mail2.sysu.edu.cn (G.H.); huangh237@mail2.sysu.edu.cn (H.H.); chenzhy49@mail2.sysu.edu.cn (Z.C.); jinglp@mail2.sysu.edu.cn (L.J.)

**Keywords:** fat mass, lean mass, bone mineral density, bone geometry, Chinese men

## Abstract

This study investigated the relationships of fat mass (FM) and lean mass (LM) with estimated hip bone strength in Chinese men aged 50–80 years (median value: 62.0 years). A cross-sectional study including 889 men was conducted in Guangzhou, China. Body composition and hip bone parameters were generated by dual-energy X-ray absorptiometry (DXA). The relationships of the LM index (LMI) and the FM index (FMI) with bone phenotypes were detected by generalised additive models and multiple linear regression. The associations between the FMI and the bone variables in LMI tertiles were further analysed. The FMI possessed a linear relationship with greater estimated hip bone strength after adjustment for the potential confounders (*p* < 0.05). Linear relationships were also observed for the LMI with most bone phenotypes, except for the cross-sectional area (*p* < 0.05). The contribution of the LMI (4.0%–12.8%) was greater than that of the FMI (2.0%–5.7%). The associations between the FMI and bone phenotypes became weaker after controlling for LMI. Further analyses showed that estimated bone strength ascended with FMI in the lowest LMI tertile (*p* < 0.05), but not in the subgroups with a higher LMI. This study suggested that LM played a critical role in bone health in middle-aged and elderly Chinese men, and that the maintenance of adequate FM could help to promote bone acquisition in relatively thin men.

## 1. Introduction

Osteoporosis is a common skeletal disorder in the elderly population worldwide. It causes a reduction in bone strength and leads to a high risk of fracture. Bone mineral density (BMD) has been widely used as a surrogate marker of bone strength in clinics and in investigations [1]. Nonetheless, the strength of bone depends not only upon its material composition, but also upon the spatial distribution of bone mass [2]. Evidence indicates that bone geometry is another vital determinant of bone strength, and the combination of the two indices markedly improves the accuracy of predicting osteoporotic fracture risk [3,4]. Quantitative computed tomography (QCT) is considered as the golden standard for obtaining three-dimensional structural measurements of bone. However, the relative high costs and high radiation doses to acquire quality images of QCT limit its use in clinical practice and research [5]. The dual-energy X-ray absorptiometry (DXA) technique has been newly adopted to measure bone structure with a shorter examination time and lower radiation exposure to patients. DXA-based hip structure analysis program can generate several bone geometric indices, such as cross sectional area (CSA), cortical thickness (CT), section modulus (SM) and buckling ratio (BR), some of which have been found to predict hip fracture risk independent of BMD [6,7]. Therefore, studies of both BMD and bone geometric properties are indispensable for the multidimensional assessment of bone strength and fracture risk.

Numerous studies have proposed that a low body weight or body mass index (BMI) is a strong risk factor for bone strength and fracture risk [8,9,10]. Body weight consists of lean mass (LM), fat mass (FM) and bone mass. A meta-analysis with around 20,000 subjects identified that both LM and FM were independent contributors to bone mass [11]. LM may influence bone by muscular contraction and gravitational loading, and FM may affect the skeleton via weight-bearing pathways and endocrine mechanisms [12]. 

LM has been reported to be favourable for bone strength in most studies [9,13,14,15], whilst no consensus has been reached on the effects of FM [15,16,17,18]. Some studies have concluded that FM is a protective factor of bone strength [15,16], but others found that it has no or even detrimental effects [17,18]. This discrepancy could be due to the differences of participants in studies, such as age, gender, hormonal status, ethnicity and body composition. It is suggested that FM had a stronger relationship with BMD than LM in postmenopausal women, but not in men and premenopausal women [13,19,20]. Gender and hormonal status may modify the influence of body composition on bone health. Recently, a study in adolescents further observed that FM was positively associated with BMD only in individuals with lower LM [21]. Results from this study may imply that the effect of FM on bone strength could be varied in individuals with different body composition.

It is not clear whether the relationship between FM and bone strength differs between men with varied LM. In addition, data about the effects of body composition on bone geometric structure are limited. Therefore, in this study, we first explored and detected the relationships of lean mass index (LMI) and fat mass index (FMI) with both hip BMD and bone geometry in 889 Chinese men aged 50 years and above, using linear regression as well as generalised additive models, and further examined the associations between FMI and bone phenotypes at different levels of LMI.

## 2. Materials and Methods

### 2.1. Study Population

This cross-sectional study was carried out as part of the Nutrition and Health Study in Guangzhou, a cohort study designed to assess the determinants of osteoporosis and cardio-metabolic disease [22]. Volunteers were recruited by sending invitation letters to residential buildings, by posting local advertisements, by giving health talks and from referrals in the local community. All subjects had resided in urban Guangzhou, China, for more than five years. Data from the first follow-up examinations, conducted between June 2010 and December 2013, were analyzed. Individuals with chronic diseases or conditions that may affect bone and mineral metabolism were excluded, including a history of metabolic bone disorder or left hip fracture, chronic medical illness, endocrine diseases like hyperthyroidism, major gastrointestinal operations, medications related to bone and calcium metabolism or drugs such as bisphosphonates, calcitonin and active vitamin D3 metabolites. A total of 889 Chinese men aged 50–80 years were then included in the analysis. The study was conducted in accordance with the Declaration of Helsinki, and approved by the Ethics Committee of the School of Public Health at Sun Yat-Sen University (Project identification code: 2012.1, 5 March 2012). Written informed consent was obtained from each participant.

### 2.2. Body Composition, BMD and Hip Geometry Measurements

Left hip bone and body composition scans were performed by DXA (Discovery W; Hologic Inc., Waltham, MA, USA). The scans were analysed with Hologic Discovery software version 3.2 (Hologic Inc., Waltham, MA, USA) by well-trained professionals. Total LM (in kg) was calculated as the difference between fat-free mass (FFM) and bone mineral content. The FMI and the LMI were calculated as the total FM (in kg) and the total LM (in kg) divided by height squared (m^2^), respectively, to remove the influence of overall body size. Calibration of the DXA machine using a phantom was carried out before each scanning session according to the manufacturer’s instructions. The in vivo coefficients of variation (CV, %) were 1.31% for total FM and 6.10% for FFM, as estimated from the duplicated measurements in 21 women and nine men (median age 60.1 years, interquartile range 57.2–67.5 years).

The hip BMD (g/cm^2^) and geometric properties were generated by the hip structure analysis software by analysing the data from the DXA scans. The narrowest region of the femoral neck (FN) was used and analysed. In the region of interest, the hip structure analysis programme provided the following geometric parameters: (1) CSA (cm^2^), an indicator of the axial compression strength; (2) CT (cm); (3) SM (cm^3^), an index of the resistance to bending and torsion; and (4) BR, an index that represents the propensity to buckle under compression [23]. Higher BMD, CSA, CT and SM values and a lower BR value may be related to greater bone strength. The CV for repeat scan precision were 1.92%, 1.55%, 2.19%, 2.99% and 4.62% for BMD, CSA, CT, SM and BR at the FN, respectively. 

### 2.3. Covariates Assessment

Data were collected by trained staff via face-to-face interviews based on a comprehensive questionnaire that covered demographic, occupational and dietary information, as well as physical activity, medical history and psychological status. Participants who smoked at least one cigarette per day or drank alcohol once a week for more than six months were defined as smokers or drinkers, respectively. Calcium supplement intake was defined as taking calcium tablets more than 30 times over the past year. A 79-item food frequency questionnaire was used to estimate the usual dietary intakes of the participants. Dietary nutrients and total energy intake were assessed based on the China Food Composition Table [24]. A 19-item questionnaire was designed to evaluate daily physical activity (metabolic equivalent, MET·h/d), excluding energy expended during all sleeping and/or sitting activities [25]. Height was measured without shoes to the nearest 0.1 cm by Kedao TZCS-4 wall mounted stadiometer (Kedao, Ningbo, China), and weight was measured with light clothing to the nearest 0.1 kg with Tanita TBF-418B Body Composition Analyzer (Tanita Corp., Tokyo, Japan). BMI was calculated as weight divided by height squared (kg/m^2^). Subjects were categorized into four groups: underweight (<18.5 kg/m^2^), normal (18.5–23.9 kg/m^2^), overweight (24.0–27.9 kg/m^2^), and obese (≥28.0 kg/m^2^) according to BMI criteria established by the Working Group on Obesity in China (WGOC) [26].

### 2.4. Statistical Analysis

All analyses were performed in SPSS (version 17.0, SPSS Inc., Chicago, IL, USA) and R (version 3.10, R Foundation for Statistical Computing, Vienna, Australia). The data were presented as mean and standard deviation (SD), median and interquartile range for continuous variables or number and percentage for categorical variables. The generalised additive model (GAM) was first used to explore the functional forms of the relationships between the bone parameters and the LMI or FMI, which could be curvilinear. The specific estimation and inference of the associations between the LMI or FMI and the bone variables were subsequently detected with multiple linear regression analysis. Three models were used in the GAM and linear regression analysis: Model I. bone phenotype = *f* (LMI, covariates); Model II. bone phenotype = *f* (FMI, covariates); Model III. bone phenotype = *f* (FMI, LMI, covariates). The quality of the GAM model and linear model was estimated by Akaike information criterion. Furthermore, the associations between the FMI and the bone phenotypes in three subgroups with varied LMI levels were analysed. The participants were classified according to tertiles of LMI. In LMI1 (<16.1 kg/m^2^) subgroup, FMI was divided into three groups: FMI1 (<4.4 kg/m^2^), FMI2 (4.5–5.7 kg/m^2^), FMI3 (>5.8 kg/m^2^). In LMI2 (16.2–17.3 kg/m^2^) subgroup, FMI was divided into three groups: FMI1 (<5.5 kg/m^2^), FMI2 (5.6–6.5 kg/m^2^), FMI3 (>6.6 kg/m^2^). In LMI3 (>17.4 kg/m^2^) subgroup, FMI was divided into three groups: FMI1 (<6.3 kg/m^2^), FMI2 (6.4–7.5 kg/m^2^), FMI3 (>7.6 kg/m^2^). Analysis of covariance was performed to evaluate the differences of bone variables amongst FMI tertiles within each LMI subgroup. Age, height, physical activity, smoking status, drinking status, calcium supplement intake, dietary calcium intake and dietary protein intake were adjusted as the confounding factors in all of the analyses. The dietary nutrient intakes were adjusted for total energy intake using a residual method. Weight was not included as a covariate because of its strong collinearity with FM and LM [27]. Collinearity was considered to exist if the variance inflation factor was greater than 5. A *p* value of less than 0.05 was considered to be statistically significant.

## 3. Results

### 3.1. General Characteristics of the Subjects

The basic characteristics of anthropometric indices, body composition and estimated hip bone strength of the studied 889 men are presented in Table 1. The age of these men ranged from 50.0 to 80.0 years with a median value of 62.0 years. The mean (SD) BMI was 24.0 (2.9) kg/m^2^. The mean (SD) values of FMI and LMI were 6.0 (1.6) kg/m^2^ and 16.8 (1.6) kg/m^2^, respectively. In this sample, the subjects had a lower consumption of dietary calcium and an adequate dietary intake of protein compared with the recommended nutrient intake (RNI) for Chinese (dietary calcium: 537.8 mg/d vs. 1000 mg/d; dietary protein: 75.7 g/d vs. 75.0 g/d) [28]. About 19.5% of the subjects had taken calcium supplements more than 30 times over the past year. Approximately 34.8% of the participants were smokers and around 17.3% were drinkers.

### 3.2. Associations of FMI and LMI with Bone Phenotypes

**Figure 1:**
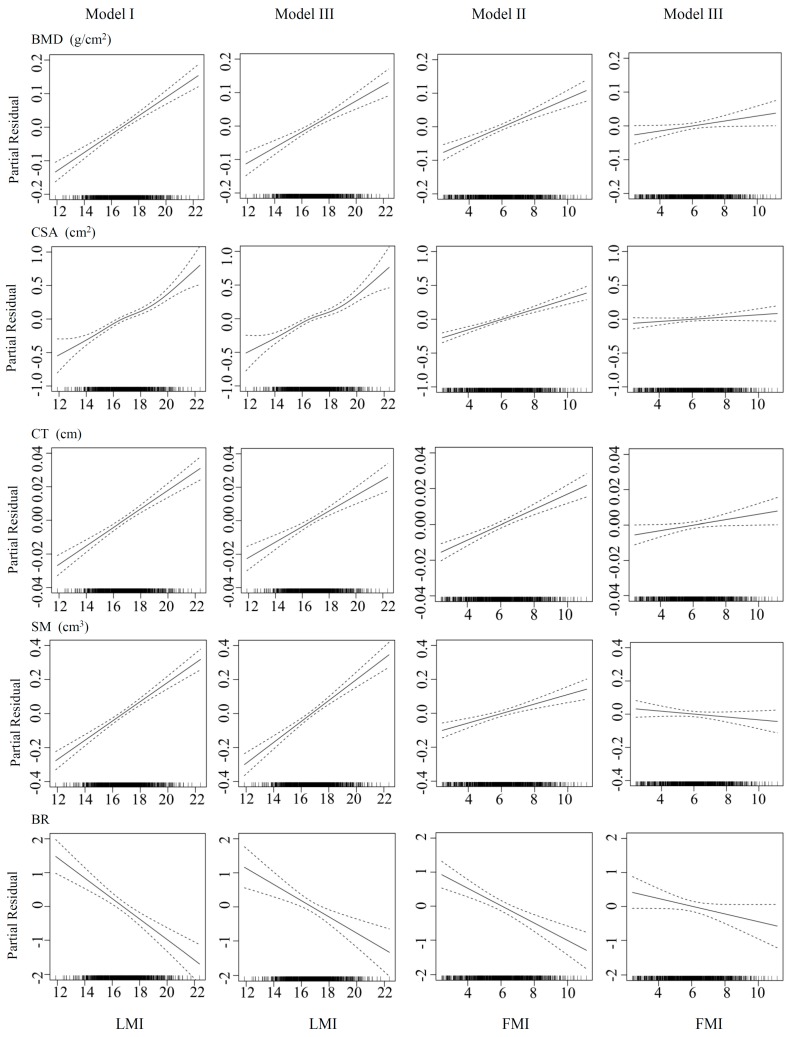
Associations between bone parameters and fat mass index (FMI, kg/m^2^) or lean mass index (LMI, kg/m^2^) using the generalised additive regression models. Dotted lines represent the 95% confidence intervals. The rug plot along the bottom of each graph depicts each observation. There were three models: I. Bone phenotype = *f* (LMI, covariates); II. Bone phenotype = *f* (FMI, covariates); III. Bone phenotype = *f* (FMI, LMI, covariates). Covariates included age, height, physical activity, smoking status, drinking status, calcium supplement intake, dietary calcium intake and dietary protein intake. BMD: bone mineral density; CSA: cross-sectional area; CT: cortical thickness; SM: section modulus; BR: buckling ratio.

Figure 1 depicts the obvious linear relationships between the FMI and all of the five studied bone phenotypes at the FN in three models. Linear relationships were also observed for the LMI with most of the bone phenotypes (i.e., BMD, CT, SM and BR). For CSA, it showed a non-linear relationship with the LMI, and its line became steeper when the LMI value was more than around 18.0 kg/m^2^. As shown in Figure 1, all of the five bone parameters altered sharply with the LMI (model I), and their associations with the FMI exhibited a less steep line (model II) after adjustment for age, height, physical activity, smoking status, drinking status, calcium supplement intake, dietary calcium intake and dietary protein intake. The LMI still maintained strong positive associations with estimated bone strength, even after the FMI was added as a covariate in the model (model III). In contrast, after controlling for the LMI, the relationships between the FMI and BMD, CSA, CT or BR became much weaker, and the positive association turned out to be negative for SM (model III).

Furthermore, linear regression analysis showed that the LMI made a greater contribution to estimated bone strength than the FMI, with the LMI explaining a higher percentage of variation in bone parameters compared with the FMI in Model III (Partial R^2^: 0.040–0.128 vs. 0.020–0.057) (Table 2). The LMI was notably associated with stronger bone, regardless of whether the FMI was adjusted (|β| = 0.005 to 0.238 in model III; *p* < 0.001). Once the LMI was taken into consideration (model III), the FMI was significantly associated only with higher BMD (β = 0.007; *p* = 0.040) and CT (β = 0.002; *p* = 0.039). No significant association was observed for the FMI and the other bone parameters. In addition, the comparisons between the GAM and the linear models showed no distinct difference.

### 3.3. Specific Associations between FMI and Bone Phenotypes within Each LMI Subgroup

**Figure 2:**
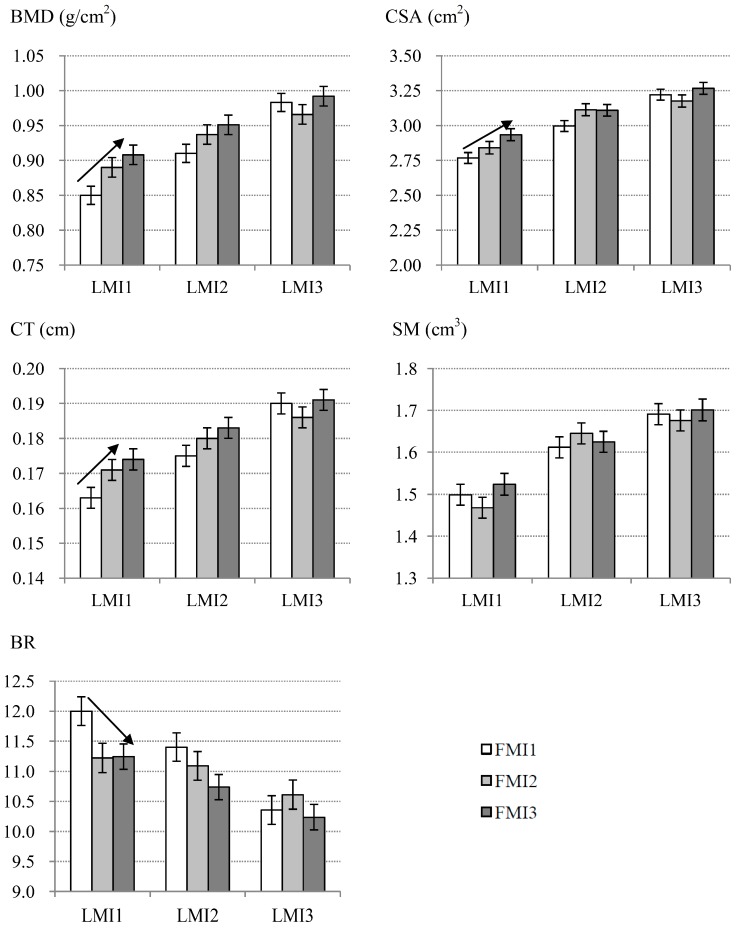
Differences of mean values of bone parameters among FMI tertiles in each LMI subgroup. Mean values were calculated after adjusting for the confounders, such as age, height, physical activity, smoking status, drinking status, calcium supplement intake, dietary calcium intake and dietary protein intake. Arrows indicate the significant trends (*p* < 0.05) for bone phenotypes with the FMI in the LMI1 subgroup.

The comparisons of the mean values of the bone phenotypes amongst the FMI tertiles within each LMI subgroup are displayed in Figure 2. BMD, CSA and CT increased and BR descended markedly with the gain of the FMI in subgroup 1 with the lowest LMI value (*p* < 0.05), but not in the other two subgroups with higher LMI values. Compared with the FMI1 subgroup, the mean values of BMD, CSA, and CT were around 6.0%–7.0% higher and the mean value of BR was about 6.0% lower in the subgroup of FMI3. No significant differences were observed for SM amongst the FMI tertiles in any of the three LMI subgroups in this study.

## 4. Discussion

In this study, we found that the LMI and FMI were positively related to estimated bone strength at the FN in Chinese men aged 50 years and above after adjustment for the potential confounders, such as age, height, physical activity, smoking status, drinking status, calcium supplement intake, dietary calcium intake and dietary protein intake. Further analyses indicated that the contribution of the LMI (4.0%–12.8%) to estimated bone strength was greater than the FMI (2.0%–5.7%). After stratification by LMI levels, FMI was correlated with stronger bone only in men with lower LMI.

Our data revealed that the LMI was a prominent predictor of estimated hip bone strength. Similar associations of whole body lean mass with hip bone geometric parameters (i.e., CSA, CT, SM or BR) were also identified in adolescent boys, males and females [9,13,29]. This result was further supported by previous findings that individuals with sarcopenia had lower BMD and an increased risk of fracture [30,31,32]. The positive effects of muscle on bone may be attributed to contraction and gravitational loading [33]. In addition, some studies have proposed that the favourable connection between bone and muscle may be also due to the endocrine function of skeletal muscle. Osteoglycin, a protein produced in myoblastic cells, plays a crucial role in bone anabolic activity. In vitro studies have shown that the mRNA levels of alkaline phosphatase, type I collagen and osteocalcin were elevated significantly in osteoglycin-overexpressed osteoblastic cells [34]. Insulin-like growth factor 1 secreted from myofibers could stimulate muscle growth and bone formation via specific receptors or insulin-like growth factor signalling pathways [35,36]. Myostatin is mainly expressed in skeletal muscle, which is also its primary target. The inhibition of myostatin has been shown to increase both muscle mass and bone mass in mice [37].

The FMI could contribute to estimated bone strength only in relatively thin men rather than those with more muscle. Studies to explore the interactive effect between FM and LM on bone strength are very limited. William et al. found a slightly stronger effect of the FMI on femoral strength index in non-obese women and men than in obese individuals [14]. In 2014, Kouda et al. investigated the independent role of the FMI to bone mass in Japanese adolescents, and their results were consistent with those of this study [21]. They found that although the FMI was significantly correlated with BMD in adolescents, the association remained positive only in the lowest tertile after stratification by the LMI. The means by which the association between the FMI and estimated bone strength is modified by the LMI is not yet clear. The complicated crosstalk of muscle, bone, and fat may be involved in the mechanism. On the cellular level, myogenic, adipogenic and osteoblastogenic lineages share a common precursor, mesenchymal stem cells, which tend to differentiate into osteoblasts and myocytes in optimal conditions and into adipocytes in unfavourable states, such as aging [38]. In addition, many cytokines take part in these interconnections. An exercise-mediated myokine, irisin, increases cortical bone mass and strength by enhancing osteoblast differentiation via the Wnt-β-catenin pathway [39]. It also activates the thermogenesis process, induces the browning of white adipose tissue and improves adipocyte metabolism [40,41]. Leptin, released from adipose tissue, was shown to have an important effect on skeletal muscle by decreasing the protein content and promoting glucose and lipid metabolism in myoblasts [42]. Moreover, it promotes osteoblast differentiation via receptor activation and inhibits bone accumulation via the central nervous system [43].

In this study, a linear relationship was found between FMI and bone in middle-aged and elderly Chinese men, which differs from some previous studies [14,44]. The inconsistency might arise from the prevalence of obesity of the studied samples. William et al. (2014), in a Canadian cohort with 40,050 women and 3600 men age >50 years, observed that the FMI showed a positive effect on bone in males with an FMI of less than 10 kg/m^2^, and in females with an FMI of less than 15 kg/m^2^ , but a negative effect in those with a higher FMI after adjusting for age, height, prior fracture, parental hip fracture, chronic obstructive pulmonary disease (COPD), recent glucocorticoid use, recent osteoporosis medication use, rheumatoid arthritis, or high alcohol intake [14]. Another study conducted in 1209 black, Hispanic and white men (age range: 30–79 years) found an inverse U-shaped association between FM and bone mineral content at the FN, with a cut point of around 30 kg with age and race/ethnicity as confounders [44]. The average values for the FMI and FM in this sample were 6.0 ± 1.6 kg/m^2^ and 16.7 ± 4.5 kg, respectively. The percentage of individuals with obesity was much lower in this sample; therefore, it was reasonable that the FMI exhibited a positive linear association with bone phenotypes in this study.

The findings from this study might be generalisable to ordinary urban middle-aged and elderly Chinese men. The results were obtained from a considerable sample size of 889 men recruited from communities in the city of Guangzhou, China, with good measurements of body composition and bone phenotypes, and with consideration of a relatively large range of confounders. This study may also have some limitations. Firstly, technical deficits in the DXA method can hardly be avoided. Nonetheless, two-dimensional data derived from DXA may describe geometric features similar to those of the three-dimensional method, as testified in previous studies [45]. Secondly, the cross-sectional design is limited in establishing a causal inference between lean mass or fat mass and bone strength. A longitudinal analysis of changes in body composition and bone strength will be conducted when the follow-up investigation of this cohort population is completed.

## 5. Conclusions

This study suggested that the LMI was more strongly associated with proximal femur strength than the FMI in Chinese men aged 50 years and above. The positive association between the FMI and estimated bone strength existed only in individuals with low levels of LMI. These findings imply that skeletal muscle may be essential to bone health in middle-aged and elderly men, and that the maintenance of adequate body fat could help to promote bone acquisition in relatively thin men.

## Figures and Tables

**Table 1:** Baseline characteristics of the studied sample (*n* = 889).

Variable	Mean ± SD/Median (25th–75th)/*n* (%)
Age (years)	62.0 (58.0–66.0)
Height (cm)	165.9 ± 5.7
Weight (kg)	66.2 ± 9.3
BMI (kg/m^2^)	24.0 ± 2.9
Obesity status	
underweight	17 (1.9%)
normal	439 (49.4%)
overweight	362 (40.7%)
obese	71 (8.0%)
Physical activity (MET·h/d) ^a^	13.9 (9.8–24.1)
Smoking status	
yes	309 (34.8%)
no	580 (65.2%)
Drinking status	
yes	154 (17.3%)
no	735 (82.7)
Calcium supplement intake	
yes	173 (19.5%)
no	716 (80.5%)
Dietary-calcium intake (mg/d)	537.8 (401.7–689.1)
Dietary-protein intake (g/d)	75.7 (59.4–87.8)
Energy intake (kcal/d)	1810.4 (1477.7–2067.0)
Percent fat mass (%)	25.5 (22.6–28.1)
FM (kg)	16.7 ± 4.5
FMI (kg/m^2^)	6.0 ± 1.6
LM (kg)	46.2 ± 5.4
LMI (kg/m^2^)	16.8 ± 1.6
BMD (g/cm^2^)	0.932 ± 0.143
CSA (cm^2^)	3.047 ± 0.478
CT (cm)	0.179 ± 0.029
SM (cm^3^)	1.604 ± 0.298
BR	10.988 ± 2.332

Data were presented as mean ± standard deviation (SD), median and interquartile range or number (*n*) and percentage (%). The lean mass index (LMI) was calculated as the total lean mass (LM) divided by height squared. The fat mass index (FMI) was calculated as the total fat mass (FM) divided by height squared. ^a^ Physical activity, excluding the energy expenditure by all sleeping and/or sitting activities, was evaluated by metabolic equivalent hours per day (MET·h/d). BMI: body mass index; BMD: bone mineral density; CSA: cross-sectional area; CT: cortical thickness; SM: section modulus; BR: buckling ratio.

**Table 2:** Associations between bone phenotypes and FMI or LMI in the multiple linear regression analyses.

Variable	Model I			Model II			Model III			Partial R^2^
	β	SE	*p*	β	SE	*p*	β	SE	*p*	
BMD (g/cm^2^)										
LMI (kg/m^2^)	0.027	0.003	<0.001				0.023	0.003	<0.001	0.088
FMI (kg/m^2^)				0.021	0.003	<0.001	0.007	0.004	0.040	0.050
CSA (cm^2^)										
LMI (kg/m^2^)	0.110	0.009	<0.001				0.101	0.011	<0.001	0.128
FMI (kg/m^2^)				0.075	0.009	<0.001	0.016	0.011	0.147	0.057
CT (cm)										
LMI (kg/m^2^)	0.006	0.001	<0.001				0.005	0.001	<0.001	0.084
FMI (kg/m^2^)				0.004	0.001	<0.001	0.002	0.001	0.039	0.048
SM (cm^3^)										
LMI (kg/m^2^)	0.057	0.005	<0.001				0.062	0.006	<0.001	0.087
FMI (kg/m^2^)				0.028	0.006	<0.001	−0.008	0.007	0.201	0.020
BR										
LMI (kg/m^2^)	−0.302	0.049	<0.001				−0.238	0.060	<0.001	0.040
FMI (kg/m^2^)				−0.254	0.050	<0.001	−0.114	0.061	0.061	0.027

There were three models: I. Bone phenotype = *f* (LMI, covariates); II. Bone phenotype = *f* (FMI, covariates); III. Bone phenotype = *f* (FMI, LMI, covariates). Covariates included age, height, physical activity, smoking status, drinking status, calcium supplement intake, dietary calcium intake and dietary protein intake. β: partial regression coefficient; SE: standard error; *p* value: associations of LMI or FMI with bone phenotypes in the linear regression analysis; Partial R^2^: variation of bone phenotypes explained by LMI or FMI.
